# Seroprevalence and detection of bovine leukosis virus in semen from breeding bulls in Costa Rica

**DOI:** 10.1007/s11250-023-03763-5

**Published:** 2023-09-30

**Authors:** Jorge Chacón, Roberto Leiva, Juan José Romero-Zuñiga, Leonel Navarro, Gaby Dolz

**Affiliations:** 1 Programa de Investigación en Andrología Animal Aplicada, Escuela de Medicina Veterinaria, Universidad Nacional, Heredia, Costa Rica; 2 Programa de Investigación en Medicina Poblacional, Escuela de Medicina Veterinaria, Universidad Nacional, Heredia, Costa Rica

**Keywords:** Bovine leukemia virus, Bull, Polymerase chain reaction, Enzyme-linked immunosorbent assay, Semen

## Abstract

Bovine leukemia virus (BLV) causes enzootic bovine leukosis, a persistent infection and the most important neoplastic disease in cattle. It is spread primarily by transferring infected lymphocytes through blood from carriers to healthy animals. The present study is aimed at determining the seropositivity of BLV in breeding bulls from Costa Rica and at detecting for the first time in the country BLV DNA in bull semen. Between May 2011 and August 2018, 379 blood and 133 semen samples were collected from bulls distributed in 118 farms. The serum was analyzed by an enzymatic immunoassay and the semen by polymerase chain reaction and sequencing. BLV seropositivity was 43.5% (165/379), while 64.4% (76/118) of the farms had positive reactors. Holstein (75.7%) and Jersey (73.0%) breeds showed the highest seropositivity. In addition, *Bos taurus* bulls (68.1%), older than seven years (50.0%), and those belonging to dairy farms (75.5%) had higher seropositivity compared to *Bos indicus* (17.7%), younger than seven years (42.2%), and those from beef farms (15.5%), respectively. Moreover, *Bos taurus* bulls had a higher risk of being seropositive than *Bos indicus* (OR = 3.4; 95% CI: 1.7–6.8). BLV DNA was found in one semen sample (2.5%; 1/40) from a seropositive bull. The importance of serum and molecular BLV screening in semen samples and the potential role of some risk factors associated with the disease, such as the bull’s age, genotype, and type of livestock productive system, is argued in the present report.

## Introduction

Enzootic bovine leukosis (EBL) is the most important neoplastic disease in cattle caused by the bovine leukemia virus (BLV), which belongs to the *Retroviridae* family (genus *Deltaretrovirus*). This viral infection causes significant economic losses in the cattle industry worldwide (Murphy et al. 1999; Sabrina 2009) and lifelong persistent infections due to the incorporation of the provirus into the lymphocyte chromosome. Most BLV-infected cattle are asymptomatic, representing a source of infection for other animals. The transmission of BLV can occur horizontally, through any biological fluid, secretions, and excretions containing infected lymphocytes, and vertically (trans placentally) in 5–10% of cases (Kerkhofs et al. 1996; Wrathall et al. 2005; Monti et al. 2007; OIE 2011). However, iatrogenic transmission seems to be the most common way of transferring BLV-infected lymphocytes to a negative animal in a herd: for example, reusing disposable syringes and not disinfecting surgical equipment between animals during livestock practices, such as tattooing, dehorning, ear tagging, and castration. Other iatrogenic routes include using a common standard plastic glove between animals during the transrectal examination. In addition, the role of hematophagous flies (*Tabanidae* family) has also been reported (Hopkins and DiGiacomo 1997; Nuotio et al. 2003; Monti et al. 2007).

Semen from infected bulls could represent a source of infection only if it contains lymphocytes, a common condition in seminal vesiculitis, and other infectious conditions from the urinary and reproductive tract (Miller and Van Der Maaten 1979; Kaja and Olson 1982). Furthermore, white blood cells (i.e., lymphocytes) can be a common finding in semen samples collected by electro-ejaculation, mainly if intrapreputial ejaculation occurs, a condition that has been recorded in about 50% of breeding bulls collected through this way (Chacón 2000). The detection of BLV DNA in semen has been reported previously. Khamesipour et al. (2013) detected the virus DNA in nine out 45 frozen semen samples from seropositive bulls. Other authors described similar results (Dus Santos et al. 2006; Sharifzadeh et al. 2011; Asadpour and Jafari 2012).

In Costa Rica, 41% (61/149) BLV seroprevalence was determined in breeding bulls from the South Pacific area (Leiva-González et al. 2018), while Beita (2008), reported in cows from dairy farms a seroprevalence of 41.0%. The BLV affects the well-being of the infected animal by predisposing it to other infectious diseases, given the impairment of its immune system. Also, the productive and reproductive parameters are negatively affected, leading to significant economic losses. According to OIE sanitary regulations, infected animals should be restricted for importation and semen trading worldwide (Chacón 2009; Asadpour and Jafari 2012; OIE 2011; González 2014; Romero et al. 2015). However, the non-regulated trading of frozen semen may be a suitable way to spread the disease into farms if lymphocytes are present in the sample (van Rijn et al. 2004). This research is aimed at reporting the BLV seroprevalence in naturally breeding bulls from Costa Rica and at determining in seropositive sires the presence of DNA BLV in their semen using molecular techniques.

## Materials and methods

### Study design and population

A transversal, descriptive, and analytical study was designed to study the BLV serological status in 379 breeding bulls belonging to 118 farms dedicated to dairy (*n* = 41), beef (*n* = 47), and dual-purpose (*n* = 30) production, distributed in the Dry Pacific and North Huetar regions, considered the two main cattle production areas of Costa Rica. No data about the BLV serological status of these farms was previously known.

The study’s authors went randomly to the farms as the owners asked for the andrological testing, a moment that was seized to sample the bulls for BLV. In addition, data regarding the sires’ breed, age, and type of farm were recorded.

Depending on the total number of bulls within each farm, between 25 and 100% of bulls were sampled. In several farms, only one or two bulls were present. Then, convenience non-probabilistic sampling was used to include the farms in the investigation. However, to appraise the BLV prevalence in bulls of the studied regions, a sample size calculation for a percentage was performed with the following parameters: 40% expected prevalence (Beita, 2008), 5% expected error, 95% confidence, and 5000 bulls’ population. Then, a sample size of 369 bulls was obtained using WinEpiscope 2.0 (Thrusfield et al., 2001).

### Blood and semen collection-handling

At least 3 ml of blood was collected from each bull (*n* = 379) between May 2011 and August 2018 from the coccygeus vessels using a coagulant-free vacuum tube (Vacutainer™ system). Immediately after collection, samples were kept at room temperature for 30 min to allow blood clot retraction, refrigerated at 4C°, and transported to the laboratory for centrifugation (10,000 g/5 min). The serum was then separated and stored at − 20 C° until analysis by an immunoenzymatic assay.

Semen was collected from 133 out of the 379 breeding bulls using electroejaculation, as described by Chacón (2000). A neat ejaculate sample (3 ml) was placed in Eppendorf® vials and stored in liquid nitrogen (− 196 C°) for transportation to the laboratory and stored until being processed by molecular technique. Only 80 semen samples were analyzed by molecular techniques due to financial limitations.

### Enzyme-linked immunosorbent assay (ELISA)

The Svanovir BLV gp51-Ab from Svanova® (Uppsala, Sweden) was used to analyze the 379 serum samples following the methodology recommended by the manufacturer. This assay reported a 100% for both sensitivity and specificity (CI 95%) (Kuczewski et al. 2018). Bull samples were analyzed in single wells using positive and negative controls in duplicates. Briefly, sera were diluted 1:25, added to the plate sensitized with BLV gp51 antigens, and incubated for one hour at 37 °C. After washing the plates three times, a conjugate (monoclonal anti-bovine IgG[H + L] peroxidase) was added, incubated for one hour at 37 °C, plates were rewashed three times, and a substrate (Tetramethylbenzidine) was added, which was incubated for ten minutes at room temperature, stopping the reaction with H_2_SO_4_. The optical density (OD), was measured using a microplate photometer at 450 nm. With the OD obtained from each sample, the positive percentage (PP) was calculated with respect to the average of the positive control using the following formula: PP = OD of bovine serum or negative control × 100/ average OD of positive control. As recommended by the manufacturer, serum samples that yielded a PP greater than 20% were considered positive if the OD of the positive controls was greater than 1.0 and the PP of negative controls was less than 15%.

### Molecular analysis

The DNA extraction from the semen samples was performed on a convenience sample of 80 bulls (40 BLV seropositive and 40 BLV seronegative), using the DNeasy Blood and Tissue Kit (Qiagen®, Chatsworth, CA-USA), following the manufacturer’s recommendations. The DNA was used to amplify a segment of approximately 385 bp of the *gag* gene of the provirus, using nested Polymerase Chain Reaction (PCR) (Wang et al. 2002). The primers that were used in the first round were BLV-F1 (5′-ATG GGA AAT TCC CCC TCC TAT-3′) and BLV-R1 (5′-GTT TTT TGA TTT GAG GGT TGG-3′); the primers used in the second round were BLV-F2 (5′-AAC ACT ACG ACT TGC AAT CC-3′) and BLV-R2 (5′-GTT CCT TAG GAC TCC GTC G-3′). The PCR reaction was carried out in a final volume of 12.5 μl, adding approximately 1-μg DNA from the sample in the first round and 2.5-μg DNA in the second round, 6.25-μl PCR Master Mix 2 × (Thermo Scientific, USA), 0.2 μm each of the two primers in each round, 1.5-mM MgCl_2_, 200-mM dNTPs, 2.5 units Taq DNA polymerase, and 11 μl of nuclease-free water. Blood DNA from a BLV-positive cow, previously confirmed by sequencing (GenBank MN315101), was used as a positive control and nuclease-free water (Thermo Scientific, USA) as a negative control. The amplification steps in both rounds were 94 °C for 3 min, 40 cycles of 94 °C for 30 s, 50 °C for 45 s, 72 °C for 60 s, and a final extension at 72 °C for 5 min. Electrophoresis of all the products of the second round was performed in 1% agarose gels, using GelRed for DNA staining and carried out in an electrophoresis chamber at 100 V for 30 to 40 min. The GenRuler 100 bp DNA Ladder Plus (Fermentas®) was used as a molecular weight marker. Bands showing a molecular weight of approximately 385 bp were considered BLV-positive and sent to Macrogen Inc. (Seoul, South Korea) to be purified and sequenced. The partial sequences obtained were edited using the BioEdit Sequence Alignment Editor® program (Hall 1999), compared with the National Center for Biotechnology Information (NCBI) database using the BLAST algorithm, and deposited in GenBank.

### Statistical analysis

Data regarding each bull, including age, breed, species, and type of livestock production system, as independent variables, were recorded with the results from the ELISA and PCR diagnostic tests (dependent variables). Fisher’s test was used to compare the percentages of positive bulls by each independent variable. Furthermore, a Kruskal–Wallis test was used to compare the bull’s age between the negatives and positives.

In addition, the epidemiological association between the serological condition of the bulls and the independent variables was carried out through an unconditional multivariate logistic regression using the ELISA result (serological status) as a dependent variable. The age of the bull, its species, and the type of livestock production system were included as independent variables. The logistic procedure was performed in two steps: univariate and multivariate analyses. All variables with *P* < 0.25 in the first step were included in the latter. A backward building model was followed based on the likelihood ratio test (Hosmer and Lemeshow, 1980). At each step of the variable exclusion process, the model tested confounding and interaction by comparison of the estimated coefficients in the new model with respect to the estimated coefficients and likelihood ratio of the old model. Confounding was judged present if at least one coefficient changed more than 0.1 when the coefficient had a value between − 0.4 and 0.4, or if at least one coefficient changed more than 25% if the coefficient had a value <  − 0.4 or > 0.4 (Romero et al., 2002). The best-adjusted model was obtained using the Akaike information criterion (AIC).

The level of significance for all calculations was 0.05. The statistical analyses were performed in Infostat® (Di Rienzo et al., 2019) and Jamovi (The Jamovi Project, 2021).

## Results

Of the 379 bulls tested, 169 (44.6%), 158 (41.7%), and 52 (13.7%) belonged to *Bos taurus*, *Bos indicus*, and crossbreeds. Regarding *Bos taurus*, Holstein (*n* = 37), Jersey (*n* = 80), and Brown Swiss (*n* = 23) were the most representative breeds, while Brahman (*n* = 118), Nellore (*n* = 20), and Gyr (*n* = 17) were the most common *Bos indicus*. An overall bull BLV seroprevalence of 43.5% (165/379) was found, with an on-farm frequency of 64.4% (76/118).

The seroprevalence of BLV was higher in *Bos taurus* and crossbred bulls than in homologous *Bos indicus* (68.1% and 42.3% versus 17.7%, *P* < 0.0001). This trend was also observed in 13 out of 22 farms where both genotypes (*B. taurus* and *B. indicus*) were simultaneously present. Similarly, the proportion of seropositive bulls was higher in those from dairy and dual-purpose farms (72.9% and 54.0%, respectively) compared to beef ranches (15.5%) (*P* < 0.0001). Regarding the bull’s age, the seroprevalence of BLV was higher in older than 3.5 years compared to younger sires (54.5% versus 31.1%, *P* < 0.0001). However, this trend was only observed in the *Bos indicus*.

*Bos taurus* bulls showed 3.4-fold times increased risk of being seropositive compared to homologous *Bos indicus* (95% CI: 1.7–6.8), while crossbreds did not differ from *Bos indicus* (OR = 1.6; 95% CI: 0.7–3.6). Regarding the productive system, males belonging to dairy and dual-purpose farms showed significantly more risk of being diagnosed as positive than bulls from beef livestock (OR: 6.3 and OR: 2.9). Finally, bulls older than 3.5 years were also more likely to be BLV seropositive than younger sires. No significant risk differences for BLV were found between other age groups analyzed. All details are shown in Table 1.

**Table 1:** Odds ratio values for BLV seropositive sires according to bull’s species, age, and type of productive system

Variables		Univariate analysis			Multivariate analysis		
		OR	95% IC	*R*^2^_McF_*	OR	95% CI	*R*^2^_McF_**
Species	*Bos indicus*	Ref		0.17			0.27
	Crossbreed	3.4	1.7–6.8		1.6	0.7–3.6	
	*Bos taurus*	9.9	5.9–16.7		3.4	1.7–6.8	
Productive system	Beef	Ref		0.20			
	Dual-purpose	4.4	2.2–8.8		2.9	1.2–7.52	
	Dairy	7.4	3.5–15.6		6.3	3.2–12.5	
Age (years)	< 3.5	Ref		0.04			
	3.5–6.9	2.6	1.7–4.0		2.8	1.7–4.8	
	7.0–10	3.0	1.4–6.2		5.3	2.1–13.2	

OR odds ratio, *95% CI* confidence interval 95%, *Ref.* reference level for OR values, *R*^*2*^_*McF*_ McFadden determination coefficient *by variable and ** total

Only one out of 80 semen samples from bulls yielded the amplicon of the expected size in PCR and was confirmed by sequencing. The positive sample was collected from a 42-month-old seropositive Holstein bull on a dairy farm located in the North Huetar area. BLAST results showed 100% nucleotide similarity (385/385 bp) with isolated BLV sequences from *Bos taurus* deposited in GenBank (AP018011 to AP18032). The sample sequence was deposited in GenBank with the accession number MN315100.

## Discussion

The prevalence of BLV-positive bulls (43.5%) is like previous reports in sires (41%) (Leiva-González et al. 2018) and dairy cows (40%) in the country (Rodríguez 1980; Mora 1997; Beita 2008; González 2014). Likewise, it is similar to data from natural breeding bulls in the USA (40.2%) (Choi et al. 2002). Other studies in semen donor bulls have reported a prevalence ranging from 5.2 to 20.9% (Dus Santos et al. 2006; Sharifzadeh et al. 2011; Asadpour and Jafari 2012).

Moreover, the frequency of BLV-positive bulls disclosed by species agrees with reports by Trainin and Brenner (2005) and Beita (2008), who found a greater prevalence of seropositivity in Holstein (*Bos taurus*) and crossbreds. These authors also reported in Holsteins a higher predisposition to develop persistent lymphocytosis, which may favor the virus spread up into the herd. Our significantly higher prevalence of positive BLV bulls in Holstein (75.7%) and Jersey (73.8%) compared to *Bos indicus* breeds (i.e., Brahman or Nellore) may suggest an inherent predisposition in the *Bos taurus* species to become BLV infected and would explain the higher frequency of positive cases found in dairy and dual-purpose farms compared to beef ones.

The possible higher risk of getting BLV infection associated with the genotype is supported by the data analysis in farms that owned both species, where they were exposed to similar management practices. In those farms, *Bos taurus* bulls had, on average, up to 35% (range 15 to 60%) of seropositivity higher than homologous *Bos indicus.* This picture resembles that reported by Petukhov et al. (2002) and Hassan et al. (2020). However, given the well-known intensive handling practices in dairy farms (Choi et al. 2002), species and management should be considered in control programs to decrease the probability of virus transmission.

The lower prevalence of positive BLV cases in bulls younger than 3.5 years agrees with Radostits (2002) and Hassan et al. (2020) and points out age as a potential risk factor for bovine leucosis virus infection. Similar results were reported in dairy cows in Costa Rica by Beita (2008).

Previous studies regarding the molecular diagnosis of BLV in semen from seropositive bulls have reported levels between 5.2 and 20.9% (Miller and Van Der Maaten 1979; Kaja and Olson 1982; Dus Santos et al. 2006; Asadpour and Jafari 2012; Sharifzadeh et al. 2011). Our survey found the BLV DNA in semen from one out of 40 seropositive sires (2.5%). Even though some authors (Gradil et al. 1999; Choi et al. 2002; Wrathall et al. 2005; Givens and Marley 2008) have stated that the risk of transmitting BLV via semen appears to be low, this statement should be carefully interpreted since it does not mean at all that semen from BLV infected bulls should be overlooked as a possible route for its transmission. Physiologically, the bull ejaculates no leukocytes unless standing as a source of white blood cells emerging from inflammatory pathologies in the reproductive or urinary tract. Then, conditions like orchitis, epididymitis, seminal vesiculitis, or urethritis, among others, may provoke the presence of lymphocytes carrying the virus and spreading the disease throughout the seminal route. In addition, the habitual presence of lymphocytes in the bull’s penis and preputial mucosa could also be a potential source of BLV transmission during natural service or even during artificial insemination with frozen-thawed semen from infected donors since contamination with white blood cells may occur during semen collection using artificial vagina or electrostimulation, especially if intrapreputial ejaculation occurs in the latest method (Chacón 2014).

Consequently, in seropositive sires where the genetic merit supports freezing its germplasm, the BLV DNA screening of their semen should be advised before its use in artificial insemination programs. Finally, the overall comparable prevalence of BLV seropositive bulls between the present and older studies in the country seems to indicate that the disease levels remain in cattle farms and that control decision rules must be enhanced in those systems to diminish the risk of spreading out the enzootic bovine leukosis.

## Data Availability

The data supporting this study’s findings are available upon reasonable request to the corresponding author.
